# Prebiotic developments

**DOI:** 10.3402/mehd.v23i0.18583

**Published:** 2012-06-18

**Authors:** Arthur Ouwehand

**Affiliations:** Active Nutrition, DuPont Nutrition & Health, Kantvik, Finland

## Abstract

Prebiotics have been defined recently more as a concept then a functional ingredient group: ‘The selective stimulation of growth and/or activity(ies) of one or a limited number of microbial genus(era)/species in the gut microbiota that confer(s) health benefits to the host’. This definition has in common with earlier definitions that modulation of microbiota composition and/or activity is its cornerstone. This has long been interpreted as an increase in faecal bifidobacteria; the so-called bifidogenic effect. However, recent regulatory evaluations have, rightfully, made clear that this is not sufficient and cannot be interpreted as a health benefit *an sich*. Therefore, a change of paradigm might be necessary.

While there is general agreement that the intestinal microbiota plays an important role in maintenance of health, there is insufficient understanding on the precise role the different components of the microbiota play here. Even the role of the microbiota in disease is poorly understood. A number of enteric pathogens is, of course, well known. However, many gastrointestinal disease or complaints have, as yet, an unknown aetiology. Thus, while it may be desirable to reduce levels of known pathogens, it is not clear what the target should be for, e.g. irritable bowel syndrome, inflammatory bowel disease, or even antibiotic associated diarrhoea (where known pathogens explain at most 50% of the cases).

Every individual has his or her own unique intestinal microbiota. It is reasonable to assume that, when a subject is in a healthy gastrointestinal state, the intestinal microbiota is optimal or close to optimal for that particular individual. Not only prebiotics but also probiotics could help in maintaining this composition when it is exposed to challenges; improving its resilience. Current molecular biological techniques make it possible to assess the full microbial composition of a faecal sample. We do not need to worry about the fact that we do not know the majority of our intestinal inhabitants or that we focus only on the groups we like (bifidobacteria?) or dislike (potential pathogens). Improving general microbiota resilience would be potentially a valid target for, e.g. prebiotics.

Another important point in the current (and previous) definitions of prebiotics is the health benefit on the host. Health benefits observed in association with consumption of prebiotics may coincide with changes in the faecal microbiota. A well-designed study will be able to unambiguously indicate the causality between the consumption of the prebiotic (or any other active ingredient for that matter) and the observed health benefit. However, it will be more difficult to substantiate a causal link with changes in the microbiota and an observed health benefit. Even when other confounding factors can be excluded, it will be challenging to unequivocally prove the causality due to the current limited mechanistic understanding of host-microbe interactions. Therefore, future prebiotic research should focus on clear measurable health benefits.

The mechanistic understanding of the interaction between the intestinal microbiota and the host and the implications of this for host health is a specific expertise that should study this. It is relevant not only for pre- or probiotics or functional foods at large but also has even implications for medicine and pharmacology.

